# Two Siblings with Familial Chylomicronemia Syndrome: Disease Course and Effectiveness of Early Treatment

**DOI:** 10.1155/2010/807434

**Published:** 2010-12-27

**Authors:** Hanan AL Azkawi, Ibrahim AlAlwan

**Affiliations:** ^1^Pediatric Endocrinology Division, King Abdulaziz Medical City-Riyadh, Riyadh, Saudi Arabia; ^2^College of Medicine, King Saud bin Abdulaziz University for Health Sciences, P.O. Box 22490, MC 3130, Riyadh 11426, Saudi Arabia

## Abstract

There are no adequate data that evaluate the safety and effectiveness of lowering triglyceride levels in very young children. The authors report a family with two male siblings, 7 and 4 years old, affected by familial hyperchylomicronemia. The oldest was diagnosed at birth during evaluation of jaundice, and the youngest showed asymptomatic hypertriglyceridemia by 6 months of age. Due to high triglyceride levels, Gemfibrozil (a fibric acid derivative) was started at diagnosis. Close clinical followup and laboratory monitoring of these children showed no side effects from the drug, and the risk of acute pancreatitis was significantly reduced.

## 1. Introduction

Hyperlipidemia is an increasingly prevalent risk factor in children, concomitant with the worldwide epidemic of obesity [1]. Lipid disorders can occur either as a primary event or secondary to an underlying disease. The primary dyslipidemias are associated with overproduction and/or impaired removal of lipoproteins. The latter defect can be induced by an abnormality in either the lipoprotein itself or in the lipoprotein receptor [2]. Monogenic disorders that cause abnormal levels of plasma cholesterol and triglycerides have received much attention due to their role in metabolic dysfunction and cardiovascular disease. While these disorders often present clinically during adulthood, some present in the pediatric population and can have serious consequences if misdiagnosed or untreated [3]. 

Hypertriglyceridemia is defined as having plasma triglyceride above the 95th percentile for age and sex [2]. It is a rare disorder in childhood. According to the National Cholesterol Education Program (NCEP), normal triglyceride level is <150 mg/dL (<1.7 mmol/L) [4]. Primary hypertriglyceridemia is the result of various genetic defects leading to disordered triglyceride metabolism. Secondary causes are acquired and may include a high-fat diet, obesity, diabetes, hypothyroidism, and certain medications (e.g., estrogen and tamoxifen) [4].

Familial chylomicronemia syndrome (FCS) is disorder of lipoprotein metabolism due to familial lipoprotein lipase (LPL) or apolipoprotein C-II deficiency (Apo C-II) or the presence of inhibitors to lipoprotein lipase [5]. It is a very rare syndrome with prevalence of approximately 1 in 1 million for homozygotes. It is relatively common for heterozygotes, approximately 1 in 500 [6]. The disease has been described in all races. To date, several hundred patients with LPL deficiency have been described [7–9]. 

FCS is the most dramatic example of severe hypertriglyceridemia. Almost all patients with fasting triglyceride levels in excess of 1000 mg/dl (11.36 mmol/L) have FCS [4]. It manifests as eruptive xanthomas, acute pancreatitis, hepatomegaly, splenomegaly, foam cell infiltration of bone marrow, and lipemia retinalis. These patients usually have lipemic plasma due to marked elevation of triglyceride and chylomicron levels [10]. 

Several mutations in the LPL gene located on chromosome 8p22 have been identified with familial LPL deficiency [11]. More than 50 missense and nonsense mutations have been identified. The majority of mutations are located on exons 3, 5, and 6 which are responsible for the catalytic coding region of the gene [6]. Apo C-II gene mutation has also been identified [12]. Other extremely rare genetic disorders can present with chylomicronemia with severe hypertriglyceridemia. Examples of these are familial apoAV deficiency, familial lipase maturation factor 1 (LMF1) deficiency, and familial GPIHDLBP1 deficiency [13]. 

We report a family with two siblings affected by FCS. We describe the clinical features, course of the disease and its management, and review the literature.

## 2. Case One

**Figure 1:**
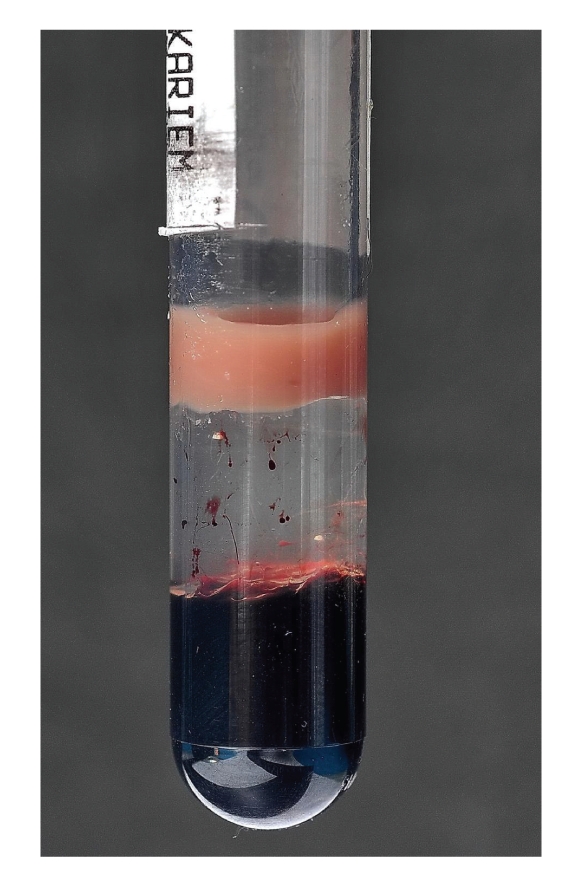
Refrigerated lipemic sample.

AA is a 7-year-old boy delivered by spontaneous vaginal delivery in a primary health care center with uneventful pregnancy. While being investigated for jaundice in the 2nd day of life, he was discovered to have high cholesterol >500 mg/dL (>5.68 mmol/L, normal <4.40 mmol/L), low hemoglobin (HB) 6.7 g/dL (normal range 13.6–19.6 g/dL), and normal serum bilirubin and platelet count. The lipid profile was repeated. The laboratory work showed a very thick blood sample that was hyperlipidemic (Figure 1). The repeated lipid profile showed (laboratory method used: AEROSET system and ARCHITECT c8000 system) serum cholesterol 7.4 mmol/L (normal <4.40 mmol/L), high density lipoprotein (HDL) 1.10 mmol/L (normal >1.55 mmol/L), and triglyceride (TG) 80 mmol/L (normal <1.70 mmol/L). Based on this very abnormal lipid profile compared to his age, the primary healthcare facility started him on lipid lowering agents: gemfibrozil (lipoid) 300 mg twice a day and pravastatin 10 mg once a day. AA was referred to a tertiary hospital at age 60 days.

Further history revealed that AA is the first child born to first-degree consanguineous parents with positive family history of hyperlipidemia, maternal side in old age (father and aunt). There is no history of sudden death, premature cardiovascular disease, or recurrent pancreatitis in the family. Both parents had no history of hyperlipidemia. 

Examination revealed an active child with no dysmorphic features or skin lesions. Abdominal examination revealed hepatomegaly. Cardiovascular examination was normal, as well as blood pressure. He was referred to the ophthalmologist for retinal examination which showed lipemia retinalis.

Laboratory investigation showed normal low-density lipoprotein (LDL), high-density lipoprotein (HDL), liver enzymes, baseline echocardiogram (ECHO), electrocardiogram (ECG), and ultrasound spleen. Ultrasound of abdomen confirmed hepatomegaly. Blood investigations are summarized in (Table 1).

Based on this incidentally discovered hyperlipidemia with very high TG and low to normal VLDL at such a young age, laboratory investigations indicate that this child probably has FCS. He was continued on Gemifirazil (lipoid) 300 mg twice a day. Pravastatin was stopped as no evidence supports its use in treating hypertriglyceridemia. The parents were referred to a dietitian and a low-fat diet was recommended, including mixing food with olive oil and giving skimmed dairy products as he is growing. Followup is ongoing.

AA was admitted twice at the age of 6 months and again at the age of 2 years. Both times he had vomiting and loose stool. He was diagnosed with acute gastroenteritis and was managed accordingly. Acute pancreatitis was suspected but could not be proved. He had no further attacks of abdominal pain or admissions and was regularly followed up. Eruptive xanthomata developed on the elbows and ear lobes at the age of four years, which was transient and resolved spontaneously. Lipid profile, liver function, and complete blood count were closely monitored (Table 1).

## 3. Case Two

SA is a 4-year-old boy. He was referred at age 16 days because of the positive family history of hypertriglyceridemia. He had full-term, spontaneous vaginal delivery with uneventful pregnancy. Examination revealed an active, but not dysmorphic baby. No skin manifestation. Abdomen showed no hepatosplenomegaly. Examination of the cardiovascular system was normal, including blood pressure. Eye examination was normal. Baseline ultrasound of abdomen, ECG, and Echo were normal. Laboratory investigations revealed TG 5.92 mmol/L, high (normal <1.70 mmol/L), cholesterol 2.32 mmol/L normal (normal <4.40 mmol/L), HDL 0.47 mmol/L (normal >1.55 mmol/L), LDL <1 mmol/L (normal <2.26 mmol/L), and HB 18.7 g/dL (13.6–19.6 g/dL). He was given dietary advice but no lipid-lowering drug was started. He was observed with close follow up.

Six months later, TG was significantly raised: TG 10.56 mmol/L (929 mg/dL), cholesterol 7.09 mmol/L, HDL 0.34 mmol/L, and LDL 1.03 mmol/L. Due to the positive family history and abnormal laboratory findings, he was started on Gemifirazil (lipoid) 300 mg twice a day. Liver enzymes were normal prior to commencing medications.

SA was also referred to a dietitian for low-fat milk and dietary advice. On follow up he was noticed to have hepatomegaly and eruptive xanthomas on the elbows, which was transient and resolved spontaneously. There was no history of abdominal pain or hospital admission for suspected pancreatitis. The eye examination showed no evidence of lipemia retinalis. Lipid profile, complete blood count, and liver enzymes were continuously monitored (Table 2).

## 4. Discussion

FCS usually manifests in childhood, but 25% of cases manifested during infancy [14] and are rarely manifest in the newborn period, as in case 1 (AA), who was diagnosed in day two of life. In India, several cases have been reported in very young children aged between 20 and 60 days. Some presented with features of sepsis with systemic complications and acute renal failure with complete recovery [15]. As mentioned above, the genetic diagnosis for FCS is available but only for limited laboratories. For our cases, the diagnosis was clinical and genetic testing was not available.

 FCS is characterized by severe hypertriglyceridemia with episodes of abdominal pain, recurrent acute pancreatitis, eruptive cutaneous xanthomata, hepatosplenomegaly, and lipemia retinalis. However, evidence suggests that presentation during infancy can be heterogeneous and may include other signs such as pallor, anemia, jaundice, irritability, and diarrhea. These manifestations are variable in the time and severity of presentation [3]. One study conducted in Quebec, Canada, in which LPL deficiency was demonstrated in 16 infants who presented with heterogeneous features, irritability, pallor, anemia,and gastrointestinal bleed, while others presented with splenomegaly and positive family history. This is also demonstrated in case one, he was accidently found to have severe hypertiglyceridemia when he was evaluated for pallor and jaundice [16].

This syndrome is autosomal recessive, and a positive family history (e.g., a child in a family) will necessitate screening of other family members (parents and siblings). Even if the lipid profile is normal, close follow up with lipid profile is indicated. In our study, the second case was screened at age one month and kept under close follow up. When his triglyceride level was significantly raised, lipid-lowering agent was commenced. The family has a 2-year-old girl who is on regular follow up for lipid profile, and her laboratory results are still within normal limits.

The most dramatic manifestation of FCS is acute pancreatitis. It is responsible for up to 7% of all cases of pancreatitis. Failure to consider and investigate chylomicronemia as a cause of pancreatitis may lead to an underestimation of incidence. Hyperchylomicronemia-induced pancreatitis rarely occurs unless triglyceride levels exceed 20 mmo/L (1760 mg/dL). Acute pancreatitis, due to any cause, is an emergency and necessitates an urgent intervention. However, in patients with hyperchylomicronemia, further management of hyperlipidemia to prevent future attacks is recommended [17–19]. 

Early diagnosis is important to prevent complications such as acute and chronic pancreatitis and pancreatic necrosis, although pancreatic function often deteriorates very slowly [3, 20]. Cardiovascular risk may also be increased in these patients, though evidence has been inconclusive [3]. Unfortunately, FCS resulting from deficiency in LPL or apo C-II is very difficult to treat with existing pharmacologic agents. The most effective treatment modality is severe dietary triglyceride restriction. The recommended targets vary from less than 50 g per day, or under 25% of total daily caloric intake, to less than 20 g per day, or under 15% [2, 21, 22]. But a significantly persistent high triglyceride level necessitates pharmacological intervention. There has been a general reluctance to use drug therapy to treat lipid abnormalities in children; however, increasing evidence suggests effectiveness and short-term safety similar to those in adults [23, 24]. Recently, the American Heart Association provides general recommendations for pharmacological management of high-risk lipid abnormalities in children and adolescents. They defined high-risk lipid abnormalities as primary and secondary conditions associated with extreme lipid abnormalities or conditions underlying high risk of cardiovascular disease whereby the presence and severity of lipid abnormalities may further exacerbate that risk [23]. 

The drugs studied and recommended for treating hypertiglyceridemia are fibric acid derivatives (e.g., Gemfibrozil, Fenofibrate). These have the effect of both raising HDL and lowering triglycerides. Main adverse effects observed were gastrointestinal upset together with an increased predisposition to cholelithiasis. Elevated liver transaminases and creatine kinase are transient. There is risk of myopathy and rhabdomyolysis-especially if used with other agents, particularly statins. Wheeler and colleagues performed a 6-month randomized cross-over trial of Bezafibrate in 14 children with familial hypercholesterolemia [23, 25]. One patient had transient elevation in liver transaminase and one patient had elevation of alkaline phosphatase. The medication was well tolerated with no impact on growth or development. Other drugs, such as stains, were also studied and found to be effective in treating familial hypercholesterolemia but did not have much effect in lowering triglyceride level [23]. Niacin is not recommended because of poor tolerance, serious adverse effects, and limited available data [1, 23, 26].

Both siblings in our study were started on Gemifibrozil 300 mg twice a day at birth and at age 6 months, respectively.

AA is now 7 years old and has had no attacks of acute pancreatitis, although he was admitted twice with acute gastroenteritis symptoms with suspicion of pancreatitis. He has also had lipemia retinalis since birth due to very high levels of triglyceride. Retinal evaluation after starting dietary and drug therapy revealed improvement. SA is now 4 years old and has never been admitted. There were no recorded attacks of abdominal pain. His retinal examination remained normal. Both children continue to tolerate Gemifibrozil very well. Triglyceride levels ranged between 11 and 25 mmol/L for AA and more than 16 mmol/L for SA. Both children have transient elevated liver transaminase, while alkaline phosphatase remains within normal limits.

Our study noted that the hemoglobin (Hb) was low when the triglyceride level was high. This was initially noticed when AA was first diagnosed. His Hb was very low at 6.7 g/dL (normal 13.6–19.6 g/dL), with no evidence of hemolysis. The Hb level improved with his improving triglyceride level. This anomaly was also seen in SA, whose Hb, when initially seen, was 18.7 gm/dL and lipid profile was mildly elevated. Later, when he started to have significantly raised triglyceride levels the Hb continuously dropped. Recent tests showed HB 10.9 g/dL normal range (11.0–14.5 g/dL) with triglyceride level >16 mmol/L (normal <1.70 mmol/L). We have no explanation for this observation.

We believe that this study is the first to report FCS in Saudi Arabia. A study was conducted in Saudi Arabia in 2001 for the prevalence of plasma lipid abnormalities in Saudi children and concluded that the hypertriglyceridemia was not seen as a major problem with only 1.96% of the total children felt to be in the high-risk group [27]. 

## 5. Conclusion

Familial chylomicronemia syndrome (FCS) is a disease of late childhood and adolescence; however, cases have been reported in infants and neonates. The syndrome presentation is heterogeneous in a very young age group. Early diagnosis and medical intervention by lipid-lowering agents and dietary modification, at the time of diagnosis, can improve the prognosis and maintain a near normal lifestyle for affected children, as the risk of pancreatitis and frequency of hospital admissions is significantly reduced. Children tolerate these agents well and show no serious side effects. Long-term studies are still needed to ensure the safety and effectiveness of these agents in children.

## Figures and Tables

**Table 1:** Results of Lipid profile, liver function, and complete blood count for AA at 2 months, 18 months, 2 years, and 4 years.

		Case 1				Normal range
		2 months	18 months	2 years	4 years	
Lipid	CHO (mmol/L)	2.58	1.7	3.99	4.4	<4.40
	TGD (mmol/L)	5.26	>25	11.32	>10.06	<1.70
	HDL (mmol/L)	0.6	0.6	0.48	0.37	<1.55
	LDL (mmol/L)	1.04	0.5	0.87	0.7	<2.260
	VLDL	NS	4.45	NS	NS	

LFT	ALK (u/L)	232	335	369	343	<500
	AST (u/L)	39	45	60→29	33	3–34
	ALT (u/L)	21	18	14	15	5–55
	GGT (u/L)	1	2	9	7	12–64

CBC	Hb (g/dL)		10.5		12.4	11.0–14.5
	Hct (l/L)		0.287		0.287	0.340–.440

**Table 2:** Results of the Lipid profile, liver function, and complete blood count for SA at 1 month, 6 months, 1 year, 2 years, and 4 years.

		Case 2					Normal range
		1 month	6 months	1 year	2 years	4 years	
Lipid	CHO (mmol/L)	2.32	7.09	5.27	4.78	3.65	<4.40
	TGD (mmol/L)	5.92	10.56	12.37	>16.05	>16.05	<1.70
	HDL (mmol/L)	0.47	0.34	0.47	0.4	0.46	<1.55
	LDL (mmol/L)	<1	1.03		0.61	0.66	<2.260
	VLDL						

LFT	ALK(u/L)	189	283	169	379		<500
	AST(u/L)	49	54	55	39		
	ALT(u/L)	21	18	38	24		
	GGT(u/L)	99	17	36			

CBC	Hb(g/dL)	18.7	13.6	12.2	10.9		11.0–14.5
	Hct(l/L)	0.549	0.39	0.339	0.334		0.340–.440

## Abbreviations

CHO: Cholesterol
TGD: Triglyceride
HDL: High density lipoprotein
LDL: Low density lipoprotein
VLDL: Very low density lipoprotein
ALK: Alkaline phosphatase
AST: Aspartate transaminase
ALT: Alanine transaminase
GGT: Gamma glutamyl transferase
Hb: Haemoglobin
Hct: Hematocrit
LFT: Liver function test
CBC: Complete blood count.
